# Supporting Fisheries Management by Means of Complex Models: Can We Point out Isles of Robustness in a Sea of Uncertainty?

**DOI:** 10.1371/journal.pone.0077566

**Published:** 2013-10-30

**Authors:** Loïc Gasche, Stéphanie Mahévas, Paul Marchal

**Affiliations:** 1 Unité Ecologie et Modèles pour l’Halieutique, IFREMER centre Atlantique, Nantes, France; 2 Laboratoire Ressources Halieutiques, IFREMER Centre Manche Mer du Nord, Boulogne-sur-Mer, France; Dauphin Island Sea Lab, United States of America

## Abstract

Ecosystems are usually complex, nonlinear and strongly influenced by poorly known environmental variables. Among these systems, marine ecosystems have high uncertainties: marine populations in general are known to exhibit large levels of natural variability and the intensity of fishing efforts can change rapidly. These uncertainties are a source of risks that threaten the sustainability of both fish populations and fishing fleets targeting them. Appropriate management measures have to be found in order to reduce these risks and decrease sensitivity to uncertainties. Methods have been developed within decision theory that aim at allowing decision making under severe uncertainty. One of these methods is the information-gap decision theory. The info-gap method has started to permeate ecological modelling, with recent applications to conservation. However, these practical applications have so far been restricted to simple models with analytical solutions. Here we implement a deterministic approach based on decision theory in a complex model of the Eastern English Channel. Using the ISIS-Fish modelling platform, we model populations of sole and plaice in this area. We test a wide range of values for ecosystem, fleet and management parameters. From these simulations, we identify management rules controlling fish harvesting that allow reaching management goals recommended by ICES (International Council for the Exploration of the Sea) working groups while providing the highest robustness to uncertainties on ecosystem parameters.

## Introduction

The Eastern Channel is a very important area because of its high biodiversity and the many fisheries it sustains [1], the most emblematic being the flatfish fishery. Sole (Solea solea) is one of the most economically valuable flatfish species in this area [2]. Fishing mortality applied to sole being high [3], there are risks that the sole population may be over-harvested. This may have critical consequences for the sole population, bycatch species and the economic viability of fishing vessels. The level of risk is highly dependent on the level of fishing effort, but also on our level of knowledge on environmental and biological parameters. For instance if natural mortality (a parameter that is usually very difficult to determine) is higher than what is commonly deemed to be the correct value, then fishing mortality is overestimated and fishing regulations will not have the expected impact.

The ICES (International Council for the Exploration of the Sea) stock assessment working groups have traditionally dealt with uncertainties by means of a precautionary approach. When possible a limit spawning biomass and/or fishing mortality are defined, beyond which the risk of recruitment impairment is high [4]. In addition, more conservative reference points have also been defined, based on a precautionary approach (PA points). These PA points aim to prevent reaching the critical limit, despite uncertainty in the ecosystem state or in the fishing effort [4]. Total Allowable Catches (TACs) should be adjusted yearly depending on the estimated state of the stock so that these limit reference points are not reached.

Even if flatfish populations in this area have been studied in depth, many uncertainties remain concerning their biology, their dynamics, or the fishing pressure they are subject to. Therefore, current management cannot guarantee that management goals will be reached. Indeed, the method currently used by ICES working groups does not explicitly take uncertainties into account and is only based on past ecosystem states, not anticipating situations that have not been observed yet. One possible way to circumvent these limitations is to model the ecosystem of interest and our uncertainties on ecosystem parameters, then to test the performance of management measures. If management measures can be found that always allow reaching management goals, then such management measures can be considered robust to uncertainties. In this paper, we propose to determine whether simple changes made to current management measures can allow reaching management goals with a higher robustness to uncertainties.

Uncertainties and risks have been increasingly taken into account in fisheries management since the beginning of the 1990s [5], [6] and now pervade modern fisheries management [7]. Many methods dealing with risks in fisheries management have been developed and have been reviewed in [8] and [7]. Most quantitative methods are based on a Bayesian approach, as advocated in [9]–[12]. This probabilistic approach is based on the use of available a priori information on the shape of probability density functions that are attributed to model parameters.

However, using probability density functions is possible only if enough knowledge is available to estimate them precisely; otherwise it only amounts to adding more uncertainties to the model. This is especially the case with complex ecosystem models where sources of uncertainty [6], [13]–[15] impacting model outputs are even more difficult to identify, as the number of modelled processes increases. In addition, ecosystems are usually complex, nonlinear and strongly influenced by poorly known ecological variables [16], [17]. Among these systems, marine ecosystems have enormous biological uncertainty [9] and exploited populations in general are known to exhibit large levels of natural variability [18]. Therefore, in some cases it may not be possible or desirable to give probability density functions to model parameter values when dealing with such ecosystems.

Choosing to not define probability density functions makes the use of methods commonly used to deal with risk less tractable. However, methods have been developed within Decision Theory that aim at allowing decision making under severe uncertainty. One of these methods is the information-gap decision theory [19], [20]. The info-gap method has started to permeate ecological modelling, with recent applications to conservation [21]–[23]. However, these practical applications have so far been restricted to rather simple models with analytical solutions and many limitations of this approach have been evidenced [24], [25].

To determine whether management measures can allow robustly reaching management goals for sole and plaice (Pleuronectes platessa) in the Eastern Channel, we build an ISIS-Fish [26]–[29] model of ICES area 7D. This spatialized fisheries dynamics model allows us to represent both fish populations and fleets targeting them, and model management scenarios. From this model, we apply a method based on decision theory so as to find out if management goals on these species can be reached despite uncertainties. First, the input parameters space of our model is explored by means of sensitivity analysis techniques. This allows us to identify and rank parameters that most influence model outputs and whose uncertainty should be tested against management measures in priority. Once enough model runs have been performed, we split combinations of model parameters between those giving output variables equal to or above our management goals and those that do not allow reaching management goals. So as to identify combinations of management parameters and ecological parameters needed to reach management goals, supervised classification is performed by means of classification trees on the dataset obtained with sensitivity analysis. This classification allows us to identify management parameter values that are most desirable so as to reach management goals, and what level of uncertainty on environmental parameters can be tolerated without compromising the achievement of management objectives.

## Materials

### ISIS-Fish

ISIS-Fish was designed to simulate and evaluate policies in the context of mixed fisheries (multi-species multi-fleet fisheries) and to take into account the spatial and seasonal heterogeneities in the distribution of resources and fishing activities [28]. This fishery model is based on three submodels: (i) a fishing activity dynamics model, (ii) a population dynamics model and (iii) a management dynamics model. Each submodel is spatially and seasonally explicit, with a monthly time step. The three submodels interact only if they overlap in space and time. The modelled area is represented by a grid, the resolution of which, in latitude and longitude, is chosen with respect to the dynamics being described and the available knowledge of the studied fishery. Within this region, zones (i.e. sets of contiguous grid cells) are defined independently for each population, each fishing activity, each management measure. Seasons are defined as sets of successive months. It is also possible to take into account fish price as well as fixed and variable costs in ISIS-Fish [27] to better model fishers behaviour. In our model, fish price is the only economic variable needed to determine the choice of fishing areas.

### The Eastern Channel

Twenty-six exploited species can be found in the Eastern Channel, but also feeding, spawning and nursery grounds, as well as migration routes. Most catches come from the French and the English fleets, the English fishing activity having decreased a lot in the past decades with only a few ports maintaining a fishing fleet on the South-Eastern coast of England. On the contrary, the French fleet in this area still comprised 641 ships in 2005 that landed more than 90000 tons of fish, worth 218 M euros [30]. The harbour of Boulogne-sur-Mer is the biggest fishing harbour in this area (ICES area 7D) with 171 active fishing vessels in 2009 [31]. The majority of landings are demersal species, especially common sole, scallops and whiting. Plaice is an important bycatch of fishing vessels targeting sole and is also directly targeted by fleets from the Netherlands and Belgium. Sole and plaice are mostly caught by beam-trawlers and netters.

Populations of sole and plaice are managed by means of TACs which build to some extent on catch limits recommended by ICES. Until the end of 2010 these catch limits aimed to keep the fishing mortality below precaution fishing mortality (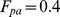 for sole and 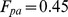 for plaice). In 2011 a transition framework to maximum sustainable yield (MSY) was implemented. This transition framework is based on a harvest control rule (HCR) spanning a 5-year period. The goal is to reduce the fishing mortality from current levels to the fishing mortality providing the maximum sustainable yield (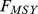). So as to reach 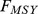 by the end of the HCR, the level of fishing mortality tolerated (and therefore the associated catch limit) is progressively decreased from 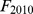 to 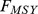. Year-to-year variation in catch limits is bounded to 15% [4].

### The Eastern Channel Model

This study is based on an ISIS-Fish model of the English Channel by [32]. This model is deterministic: a given set of parameter values always gives the same values for the output variables. We performed 10-year simulations so as to model a 2008–2017 period that encompasses the 2010–2015 period of the ICES transition framework to MSY. This allows us to force some input parameter values to their estimated value for the first three years modelled (2008–2011) and then test transition scenarios and determine their consequences on the ecosystem.

#### Exploitation

This model focuses on the French flatfish fishery. Only French gillnetters and English or Belgian beam trawlers are explicitly taken into account and the modelling assumption is made that the travelling time from their home harbour to fishing grounds is negligible. It is considered that they fish all year long in ICES areas 7D and 7E and fleet (i.e. group of boats with same characteristics belonging to the same harbour) parameterisation is the same for both areas. They target sole (Solea solea) and their main bycatch is plaice (Pleuronectes platessa). An important notion when defining fishing activities in ISIS-Fish is that of métiers. For a given fishing vessel and a given month of the year, the métier practised by the vessel is defined by the gear used, the target species and the fished area [33], [34]. Some fishing units have the same métier all year long, others change of métier (i.e. change of fishing area and/or gear and/or target species) depending on the season. A succession of métiers in a year defines a fishing strategy. Métiers, as well as entire fishing strategies can be common to groups of vessels. Fishing units with the same fishing trip duration, the same number of trips per month and belonging to the same harbour belong to the same fleet [28]. Notions of fleet and métier are not totally correlated; a given métier can be common to ships belonging to various fleets. Métiers, fishing strategies as well as fleets are defined based on real data (e.g. commercial logbook data, fishers interviews, observer data, etc.), the level of detail depending on the available information and the modeller’s needs [29]. In our model métiers parameterisation differs between ICES area 7D and ICES area 7E, but in general trawlers target both species with almost the same intensity while netters clearly focus on sole. These two species are the only ones explicitly represented in the model, other species caught by this fishery being grouped together in a single group. Our goal being to study flatfish fisheries, we are mostly interested in the Eastern Channel (ICES area 7D). However, fishing activities and fish stocks in the Eastern and Western part of the Channel being linked, we chose to model both areas and emphasize results obtained on the Eastern Channel. In our model, fishers select their métier dynamically by means of a gravity model. The attractivity of each choice is estimated yearly from fishing habits and past outcomes of the fishery [27]. Once the catch limit has been reached for a species a set of conditions are applied in an attempt to realistically model fishers’ behaviour: for a given métier i) if the species only is a bycatch species then fishing goes on and this species is discarded, ii) if the species is the target species then the métier stops and fishers look for another métier for the remaining months of the year. The choice of an alternative métier depends on the ease of implementation of the métier: métiers within the same strategy (i.e. monthly choice of métiers for a fleet during the year) using the same gear are preferred to métiers where a change in fishing gear is needed or métiers outside the strategy (that can correspond to no fishing activity). Discarded fish have a chance to survive that is species-dependent but age-independent and discarded fish that survive are returned to the abundance of their year class.

#### Populations

Both species are assumed to be distributed homogeneously over the whole modelled area. Each species is split in two populations, one for area 7D and one for area 7E. Biological parameters, and in particular weight-at-age, maturity, initial fish abundance, correspond to those estimated for year 2008 by the ICES Working Group on the Assessment of Demersal Stocks in the North Sea and Skagerrak (WGNSSK) [35]. Fish catchability was calibrated so that, for each population, fishing mortality at age for year 2008 in our ISIS-Fish model corresponds to that estimated by the working group for year 2008 in the 2011 stock evaluation. In our model, the spawning biomass of sole in the Channel seems to be within acceptable biological limits, but with a high fishing mortality. No reliable stock recruitment relationships could be fitted to these stocks. We used spawning biomass precautionary thresholds (as suggested by [14]) that were defined by the ICES. We forced reference values of recruitment for years 2008, 2009 and 2010 (Table 1) to values estimated by ICES [35]. From year 2011 onwards, recruitment was fixed as the geometric mean of past recruitment values [35].

**Table 1:** Recruitment (in number) values used for the first three simulated years and after.

PopulationRecruitment	2008	2009	2010	after
Sole 7D	2.395e7	5.298e7	2.817e7	2.353e7
Plaice 7D	1.157e7	2.343e7	1.498e7	1.216e7
Sole 7E	2.379e6	2.885e6	4.301e6	4.301e6
Plaice 7E	5.560e6	1.006e7	5.007e6	5.007e6

#### Management

We chose to focus only on the current transition scenario to MSY and to test a wide range of values for parameters defining this management scenario. This corresponds to situations where managers have already chosen how to manage a resource or an ecosystem, but where uncertainties remain on the best way to apply the chosen scenario. This allows us to find which particular range of parameter values gives maximum efficiency to the management measure. It also permits determining whether a range of parameter values allows reaching some robustness to uncertainties on biological parameters.

For the first three years of simulation, populations are managed using TACs. TAC values for these years correspond to those that were applied in 2008, 2009 and 2010 (Table 2). As a single TAC level was used to manage plaice in areas 7D and 7E, this TAC was split so that a TAC level could be attributed to each population. Plaice TAC in area 7D was set to 3500 t for years 2008 and 2009 and to 3400 t for year 2010, as recommended in ICES stock evaluations [3], [36], [37]. The remaining part of the total TAC was attributed to plaice in 7E. This allowed us to get 2010 TAC levels for all simulated populations and simulate harvest control rules thereafter. From 2011 onwards TAC values are determined for each population by a harvest control rule (HCR) that controls the transition towards MSY. Every year during the transition period a value of maximum fishing mortality to be applied to the ecosystem is computed as a combination of the 2010 fishing mortality and the fishing mortality that would give the maximum sustainable yield. Transition duration being 5 years the proportion of 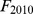 in the computed F decreases by 20% every year and the proportion of 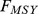 increases by 20%. The TAC level computed by the HCR is determined by other conditions in addition to those on fishing mortality: (i) Spawning biomass has to be above a minimum level (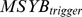), (ii) TAC value cannot change by more than 15% from one year to the next, and (iii) the computed fishing mortality has to be below 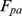. A minimum landing size is also implemented for each species: 27cm for plaice and 24cm for sole.

**Table 2:** TAC values used for the first three simulated years.

TAC Population	2008	2009	2010
Sole 7D	6593t	5274t	4219t
Plaice 7D	3500t	3500t	3400t
Sole 7E	765t	650t	618t
Plaice 7E	1550t	1146t	874t

The database used in this paper, including HCR and gravity model java code, can be downloaded from the ISIS-Fish website (http://www.isis-fish.org/download.html), as well as the latest version of the ISIS-Fish model.

## Methods

### Decision Theory

The info-gap decision theory [19], [20] aims at allowing decision making under severe uncertainty. This theory allows comparison between various courses of action (q 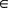 Q), depending on states of Nature (u 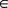 U). “u” is called the “ambient uncertainty” in the info-gap theory. The reward function 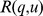 gives the expected outcome for a given course of action q and a given state of Nature u. When using a model to compute 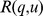, the model gives the value of 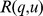 associated to parameter values used to perform the simulation. The decision maker has to choose a critical value (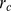) below which the reward function should not drop (in case a high value of the selected output variable is desirable, for instance a high fish biomass). The robustness function (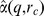) is the greatest horizon of uncertainty 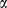 that can be tolerated (on Nature's state or on variables controlled by human activities) while being sure that the reward function did not cross 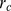. The Info-Gap theory proposes several functions of uncertainty to compute the robustness function. In practise, the most popular one consists in testing parameter values around the reference parameter values set in the model that increasingly differ from these reference values, until a combination of values is reached for which 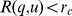. The distance between the last parameter values tested for which 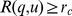 and the reference values corresponds to 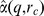. As a result, the decision maker knows for each possible action and state of Nature the level of uncertainty that can be tolerated. Thus, it is possible to choose the action that seems most appropriate to the situation, depending on management goals, local knowledge of the fishery and the environment, and the level of risk that is accepted by stakeholders. If the probability of occurrence of the various ecosystem states is not known, then a minimax approach [38] or other aspects of the Theory of Games [39] can be applied.

Many limitations of the info-gap approach have been underlined by Sniedovitch [24], [25] and are reviewed in [40]. One of these is that it is performed around a given reference point and therefore is inherently local and not suitable to situations of severe uncertainty. This particularly is an issue in fisheries science where reference model parameterisations (corresponding to our knowledge of the state of an ecosystem) often poorly meet management goals. When this happens, then 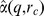 is very small, and many other values of the parameters could lead to acceptable reward R. So this approach seems to be very conservative and cautious and does not fully allow the exploration of the input parameters space and the identification of an area in the input parameters space that gives acceptable results. Therefore, we choose to define a priori the window in the input parameters space that we wish to explore and perform the exploration by means of exploration techniques from sensitivity analysis.

### Applying Info-gap to a Complex Model

To our knowledge, the Info Gap Theory has only been applied to analytical models. In the context of complex models (with no analytical solution), deriving the robustness function is a great challenge. The ISIS-Fish model belongs to this family of complex models. The model can be used to simulate the reward function 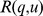 for each selected couple (q,u) in QxU. Many model runs have to be performed to explore the robustness of R to uncertainty in q and u. Therefore, we propose a pragmatical approach to apply Info-Gap theory to complex model, following two main steps: 1) a sensitivity analysis of the simulation model performed on the input parameters space UxQ; 2) a classification trees analysis fitted to the model's outputs (simulated for the previous step) to discriminate the sensitive parameters and their range of variation accounting for reward 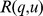 above the critical value 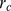.

#### Perform sensitivity analysis

A good exploration of the input space can rely on the powerful tools provided by sensitivity analysis techniques. Many sensitivity analysis techniques are available to modellers [41], [42].

Most sensitivity analysis techniques can be divided into two parts: a method to explore the parameter space and a method to rank parameters according to their levels of sensitivity. Following [43], [44] we chose to focus on a global sensitivity analysis method associated to a variance decomposition method, instead of a one-at-a-time (OAT) method. The difference between local and global sensitivity analysis techniques is that global techniques study variations of the output variable over the entire range of values of the input parameters [45], [46]. The main asset of global sensitivity analysis is that it allows us to measure interaction effects, which can be of great importance in complex ecosystem models.

**Figure 1:**
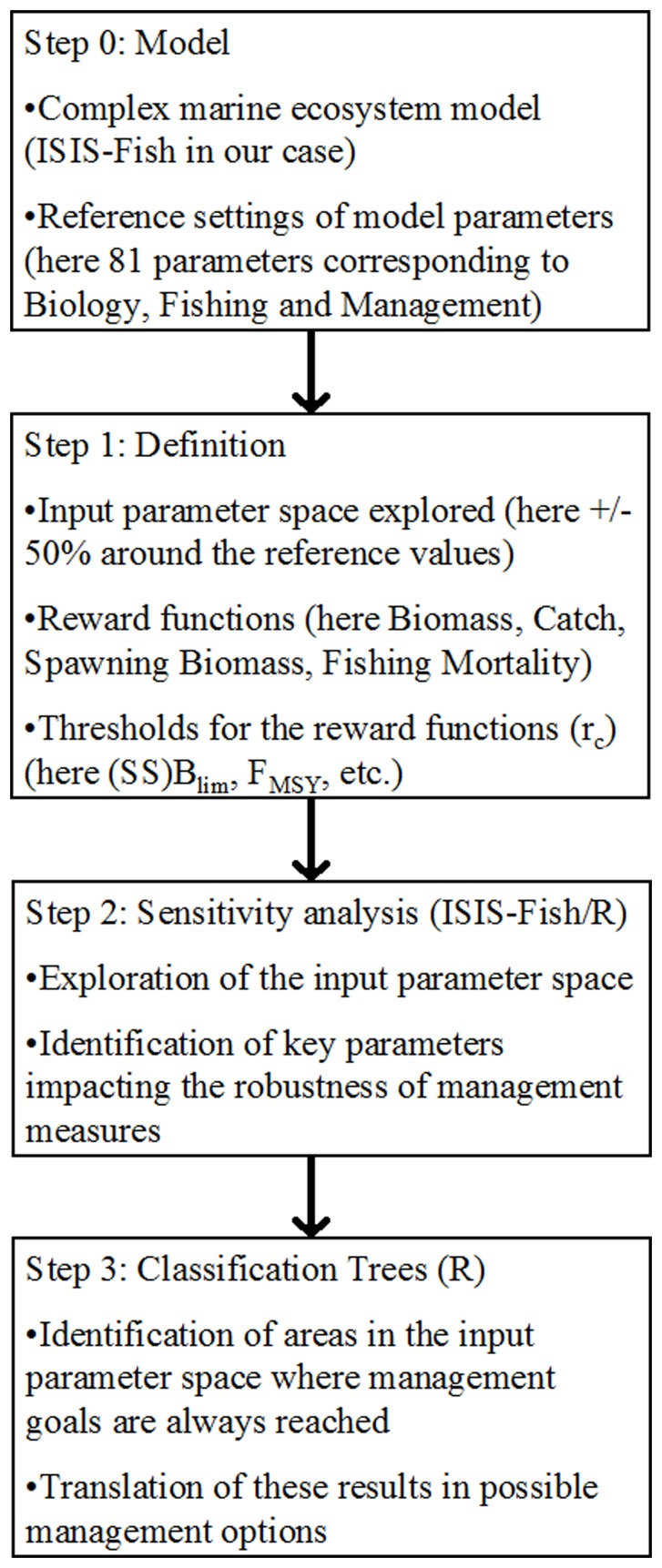
The general approach used to identify areas of interest in the input parameters subspace. Once the model has been built and parameters identified (step 0) output variables to be studied and thresholds corresponding to these variables can be chosen and the input space to explore defined (step1). Then the input parameter is explored and important parameters identified (step 2). Classification trees are used to classify input parameters values depending on the output values they gave when exploring the input parameter space (step 3).

We identified 81 parameters from our model on which to perform sensitivity analysis (Fig. 1,Step 0). These parameters can be split into three groups: biological parameters, technical parameters and management parameters. Biological and technical parameters (Table 3) correspond to states of Nature with various levels of uncertainties and management parameters (Table 4) allow us to test various management scenarios.

**Table 3:** Biological and technical parameters tested, for every population, métier or gear.

Parameter Name	Abbreviation
Catchability	Q
Mean Weight	MW
Recruitment	RE
Natural Death Rate	NDR
Growth Rate	K
Asymptotic Length	Linf
Time at the Origin	T0
Price	P
Selectivity Beam Trawl	SBT
Selectivity Net	SN
Selectivity Other Gears	SO
Target Factor Beaming	TFB
Target Factor Netting	TFN
Target Factor Other Métiers	TFO
Proportion of effort allocationcoming from habits	habit

50% variations were tested around the reference value. Each biological parameter exists in five versions, one for each population: sole 7D (S7D), plaice 7D (P7D), S7E or P7E and one for the “Other” group. Technical parameters are either defined at the population scale (S7D, etc.) or at the area scale (7D, 7E, or both: 7DE).

**Table 4:** Management parameters tested.

Parameter Name	Abbreviation	Reference Value
Minimum Landing Size for Sole	MinSizeS7DE	24cm
Minimum Landing Size for Plaice	MinSizeP7DE	27cm
Duration of the transition framework	Trans	5yrs
Survival rate of discarded fish	PropSurv	0.25
Maximum yearly TAC variation	varTAC	0.15
Targeted fishing mortality at MSY, Sole 7D	FmsyS7D	0.29
Targeted fishing mortality at MSY, Plaice 7D	FmsyP7D	0.23
Targeted fishing mortality at MSY, Sole 7E	FmsyS7E	0.27
Targeted fishing mortality at MSY, Plaice 7E	FmsyP7E	0.19
Precautionary fishing mortality, Sole 7D	FpaS7D	0.4
Precautionary fishing mortality, Plaice 7D	FpaP7D	0.45
Precautionary fishing mortality, Sole 7E	FpaS7E	0.4
Precautionary fishing mortality, Plaice 7E	FpaP7E	0.45
HCR Trigger Biomass, Sole 7D	MsyBtS7D	8000t
HCR Trigger Biomass, Plaice 7D	MsyBtP7D	8000t
HCR Trigger Biomass, Sole 7E	MsyBtS7E	2800t
HCR Trigger Biomass, Plaice 7E	MsyBtP7E	2500t

50% variations were tested around the reference value, except for the survival rate of discarded fish. Survival rates from 0 (no survival of discarded fish) to 0.5 (survival of 50% of discarded fish) were tested because of large uncertainties on this parameter and because the reference model value is 0. Parameters “Trans”, “PropSurv” and “varTAC” have similar reference values for all populations so they are only given once.

We chose to explore a window corresponding to +/−50% of the reference value of each parameter (Fig. 1,Step 1). This range of values agrees with observations from [47] who noted that managers can rarely measure stock levels accurately and typically use confidence intervals of 50%.

From a biological point of view, exploring the same range of values for all parameters makes little sense. This window probably does not allow us to take into account the total variability of all parameters, as some may naturally vary within a greater range, but should be wide enough to contain most variability. On the other hand, this window may be too wide for some well-known parameters with little natural variability. This has to be taken into account when studying sensitivity analysis results. Indeed, some parameters may be identified as important because an unrealistically wide range of values was tested for them. By contrast, other parameters may be identified as little impacting only due to a too narrow range of values tested in the analysis.

The selected window in the input parameters space was explored by means of Latin Hypercube Sampling (LHS, [48]) using the “sensitivity” package [49] from R [50] (Fig. 1,Step 2). Latin Hypercube Sampling is a probabilistic sampling procedure that incorporates many of the desirable features of random sampling and stratified sampling [51]. Then a a variance decomposition method gave us for each input parameter 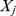 a coefficient 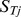,corresponding to 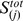 in [52], indicating whether 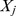 is a sensitive input parameter or not for the output variable studied. Parameters with 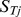 close to or equal to zero can be removed from the uncertainty analysis as they do not influence the model output. Model outputs we studied are biomass, spawning biomass, fishing mortality and catch. Spawning biomass and fishing mortality were studied in priority as they are variables commonly studied by ICES working groups. Classification trees were then built on management parameters and natural parameters most influencing model outputs.

#### Point out isles of robustness with classification trees

Once the input parameters space has been explored, one has to find the boundary between input parameter values leading to acceptable outputs (relative to management goals) and input values leading to failures (Fig. 1,Step 3). As we use a complex model to compute values of 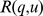 this boundary cannot be found analytically, but has to be identified from a limited number of simulations. Very powerful methods have been developed for machine learning that allow identifying the hyperplane separating input values from a dataset in two (or more) groups depending on resulting outputs, but these method are either black boxes or provide results that are too difficult to interpret for our needs. Therefore we chose to focus on classification trees [53], that allow for simpler representations of results by means of successive univariate splits of the set of input parameter values.

**Figure 2:**
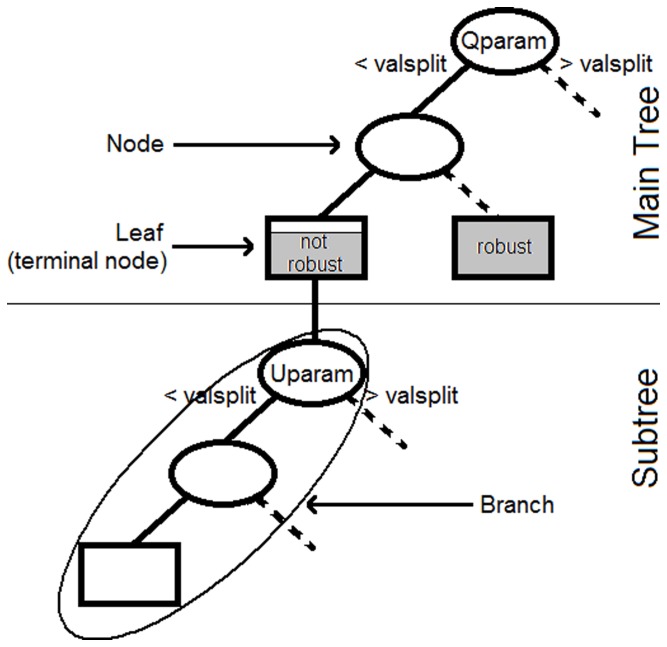
Successive use of trees for input parameters space exploration. The first tree (called the main tree) is built on management parameters only, as they are the main concern in the study. When management parameters do not allow robustly reaching management goals, trees (called subtrees) are built for each leaf of the main tree. The second set of trees is built on parameters identified as important by the sensitivity analysis. Results of interest are parameters values corresponding to branches (either of the main tree only or both the main tree and a subtree) leading to robust leaves.

A classification tree is built step by step. At each step, a split is performed on a parameter belonging to the set of parameters on which the tree is built. A split separates the dataset (values of 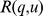 simulated by the model and associated parameters) into two parts according to values of this parameter. When building a tree, the rule to perform a split is that some measure of discrepancy between the two datasets given by the split is maximized. Therefore the tree is built from the most important node (i.e. combination of a splitting parameter and a splitting value for this parameter) to the least important one. Classification stops when some criteria defined by the user are reached. For instance, classification can stop when the information gain given by a split (i.e. the increase in discrepancy between the two datasets obtained) is lower than a defined threshold. The terminal nodes of a tree are small datasets that are parts of the initial dataset on which the tree was built. They are called leaves. For a given tree, the path leading from a node (usually the first node) to a leaf is called a branch of the tree (Fig. 2).

Our goal being to determine the extent to which management measures can allow reaching management goals robustly, we perform classification in two successive steps for each output variable (Fig. 2). Let 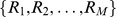 be the set of all output variables studied. Let Q be the set of all management parameters in the model: 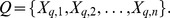 Let U be the set of parameters corresponding to important states of Nature (fish biology and fleet characteristics) identified at step 1: 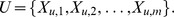

1. A classification tree (the main tree) is built based only on management parameters belonging to Q. Each leaf of the tree may contain either successes (values of 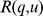 above some threshold) or failures, or a mix of both. A leaf can be considered “robust” when it contains only successes, i.e. all realisations belonging to this leaf correspond to successful management configurations, whatever the state of Nature. In practice, as the classification relies on model simulations only robust nodes with a high weight (i.e. containing many simulations) should be considered. The concept of robustness can be adapted, depending on the willingness of managers to tolerate risk, so that a node may be considered to be robust if its proportion of successes is above some threshold. Here, we fixed that threshold to 99% of successes as node boundaries can sometimes be difficult to identify very accurately and therefore a few failures can be included in a node that would otherwise be robust. At the end of this first step, leaves are classified into two classes: “robust leaves” if leaves are identified within the tree based on management that allow always reaching management goals or “not robust leaves” for others. Leaves that are not robust are used to perform a second stage of classification.
2. For each leaf that is not robust, we performed a second classification on parameters belonging to U. This allows us to grow subtrees in a limited amount of time while knowing that important parameters are tested. If a robust terminal node is identified within a subtree associated to a leaf of the main tree, then we know within which range of values of 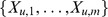 management parameter values corresponding to that leaf will allow reaching management goals.

This approach has two assets: (i) if management parameters tested are consistent with current management measures then we know what level of uncertainty or variability on natural parameters can be tolerated while still reaching management goals at the end of the period; (ii) if current management parameters do not correspond to those identified by the tree then we know how (and to what extent) current management should be altered to have a chance to reach management goals considering the uncertainty on management parameters.

The method we chose to build classification trees is that of conditional trees [54] that allows overcoming usual problems of possible overfitting, selection bias, or input parameters scaling.

We assessed tree and subtrees instability (i.e tree structure changing when slightly modifying the dataset used to build it) by means of re-sampling techniques. For the main tree and subtrees corresponding to leaves of the main tree, we built sets of 500 trees with subsets containing 95% of the dataset. From all these trees, we identified the tree appearing most often and focused on it, making the hypothesis that all trees would converge to this tree type provided the dataset is big enough. We also compute average splitting values and standard deviations from the 500 replicates so as to have clear indicators of tree variability (see Supporting Information S1 for more details about this method). If tree variability is too high (it is especially the case for subtrees, as they are built from a subset of the main dataset corresponding to their associated leaf), it makes little sense to focus only on one particular tree type. In this case, results from the most common tree types can be provided.

## Results

### Sensitivity Analyses

**Figure 3:**
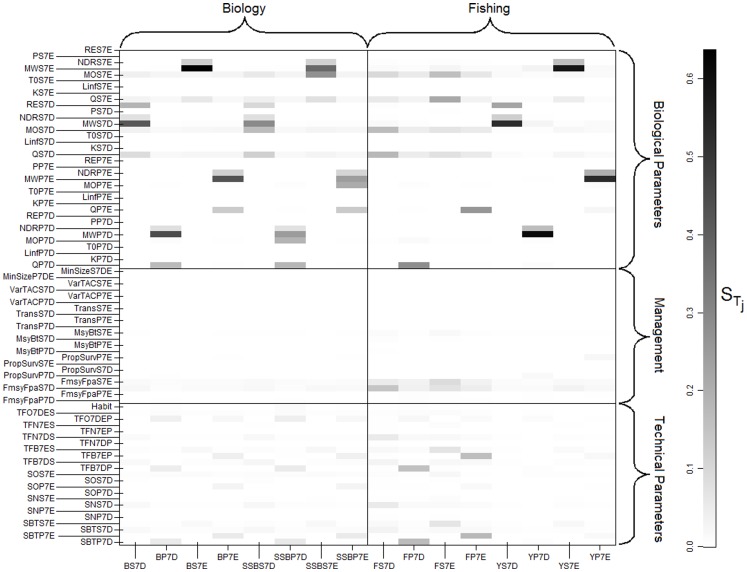
Results of the sensitivity analysis. Each row corresponds to one of the 81 input parameters tested and each column to an output variable. Output variables are biomass (B), Spawning Biomass (SSB), Fishing mortality (F) and Catch (Y). Results have to be studied in columns, black cells indicating important parameters for a given output.

Results from the sensitivity analysis are presented in Fig. 3, where each column stands for an output variable and each row for a different input parameter. Only rows should be compared as the intersection of a row and a column represents the amount of the total variance of a particular output variable explained by an input parameter. The naming of the various input parameters appearing in Fig. 3 is detailed in Table 3 and Table 4. Parameters are presented individually in Fig. 3 whereas parameters with similar values for all populations appear only once in Table 3 and Table 4. Besides 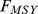 and 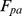 were varied jointly in the sensitivity analysis and appear as a single parameter 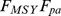 in Fig. 3.

The goal being to reduce the model, we focus only on the most sensitive parameters that really stand out compared to the others. These input parameters are listed from the most important to the least important in Table 5 and Table 6. First order interactions between parameters were also tested. Interactions are pretty straightforward, the most sensitive parameters producing the strongest interactions. Interactions do not especially drive output variables in our model. Fishing mortality is the output variable most impacted by interactions, but even in this case the most sensitive interaction is always less than 0.1 times as impacting as the most important parameter. We therefore focus only on main effects when presenting our results. It is interesting to note that the most important parameters impact the four studied populations whereas less important parameters are specific to only some of these populations. Also, small interactions between areas 7D and 7E appear, as sometimes populations from one area can be slightly impacted by parameters defined for the other area.

**Table 5:** Input parameters most impacting output variables.

Biomass				Spawning Biomass			
Mean Weight (MW)				Mean Weight			
Catchability (Q)				Maturity Ogive (MO)			
Natural Death Rate (NDR)				Catchability			
				Natural Death Rate			
S7D	P7D	S7E	P7E	S7D	P7D	S7E	P7E
RES7D	SBTP7D	MOS7E	SBTP7E	RES7D	SBTP7D	QS7D	SBTP7E
MOS7E	TFB7DP	QS7D	TFB7EP				
0.751	0.742	0.825	0.716	0.676	0.669	0.786	0.666

Parameters are sorted from the most impacting to the less impacting, and the proportion of total output variance explained by these parameters is given at the bottom of each column.

**Table 6:** Input parameters most impacting output variables (continued).

Fishing Mortality				Catch			
Catchability				Mean Weight			
				Natural Death Rate			
S7D	P7D	S7E	P7E	S7D	P7D	S7E	P7E
MOS7D	SBTP7D	MOS7E	SBTP7E	RES7D	MWS7E	QS7E	QP7E
FmsyFpaS7D	TFB7DP	FmsyFpaS7E	TFB7EP	MOS7E	MWS7D	FmsyFpaS7D	PropSurvP7E
MOS7E	MOS7E	QS7D	MOS7E	FmsyFpaS7D	PropSurvP7D	QS7D	TFB7EP
QS7E	QS7D	FmsyFpaS7D	QS7E				
0.571	0.637	0.542	0.629	0.847	0.805	0.799	0.747

Parameters are sorted from the most impacting to the less impacting, and the proportion of total output variance explained by these parameters is given at the bottom of each column.

The first thing that stands out from these results is that all studied output variables except fishing mortality are mostly impacted by biological parameters, technical parameters having a lower impact on the outputs. Management measures have little impact on all output variables, even those directly related to fishing. The parameter of our harvest control rule that most influences output variables is the target value of fishing mortality 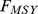 (and the associated 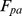). However the effects of the 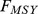 parameter are limited to fishing mortality and effects of management on biomass or spawning biomass are low.

Interestingly, while fishing mortality is driven by catchability and technical parameters, catch appears to be impacted almost only by biological parameters. Fish mean weight-at-age alone explains up to 60.0% of the total variance of the catch which leaves little variance to be explained by other parameters. This importance of fish weight may be linked to the fact that we study biomass and catch in tons, and not abundances or catch in numbers. Besides fish mean weight-at-age is a parameter with relatively small variability, and testing 50% around this parameter's reference value may artificially give it an important weight.

Mean weight is the input parameter most impacting model output variables, and is followed by natural death rate and catchability (Table 5 and Table 6). Recruitment has noticeable impact only for sole in area 7D, and this impact is moderate.

In our model, beam-trawling is the most impacting fishing activity, with high 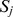
s for both its target factor (TFB) and selectivity (SBT). It impacts both populations of plaice, as well as fishing mortality of Sole in area 7E. The effects of netting are limited to sole in area 7D and can be noted only for fishing mortality (moderate 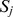
s for SNS7D and TFN7DS). Biomass and spawning biomass of plaice in area 7D are also impacted by the target factor of the “other métiers” group of our model, that bundles together all the lesser operated métiers.

### Conditional Tree Analysis

Conditional trees can be built according to the previously presented method for all output variables studied in the sensitivity analysis. However, only the most important variable for conservation, spawning biomass, will be discussed here for populations of sole and plaice in area 7D. The management goal, for both sole and plaice, is that spawning biomass must remain above 8000t.

#### Sole 7D

**Figure 4:**
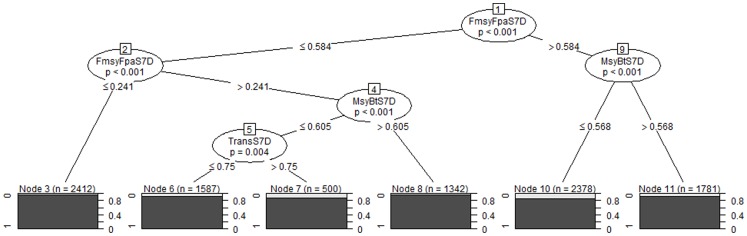
Main tree built on management measures for sole in subarea 7D. The criterion separating successes from failures is a 8000t spawning biomass threshold. Splitting variables (and values) are sorted by importance, from the top to the bottom of the tree (the most important corresponding to node 1). Values appearing on branches of the tree are the splitting values. The black and grey squares at the bottom of the tree are the terminal nodes or leaves.

The tree structure obtained when building the main tree (Fig. 4) on the entire training dataset corresponds to that identified when creating a large number of trees on a smaller subset (see Supporting Information S2 for more details about the tree-building process). Therefore we can infer that our dataset is big enough to ensure good tree stability and that all trees built from subsets would converge to that particular tree provided we had enough data. As no leaf of this tree is robust, i.e they all contain some failures, a subtree was built for each of the six leaves of the main tree to determine if management measures allow reaching management goals within a certain range of values of natural parameters, as shown in Fig. 2. Among all subtrees corresponding to a leaf of the main tree, only some subtrees are of interest (robustness+high weight), we therefore decided to focus on those. For each subtree, we compute mean splitting value and standard deviation around these values because there can be variations in the splitting values at each inner node. Branches of interest can be identified within subtrees that lead to leaves that are robust and have a high weight. Besides, it appears that these particular branches are much more stable than other branches of subtrees. Table 7 shows for each leaf of the main tree built on management measures parameters and split values corresponding to interesting subtree branches that would allow robustly reaching management goals. A very interesting thing to observe is that for all subtrees all important branches identified correspond to boundaries on natural parameters that contain the reference model parameterisation (which corresponds to value 0.5). Therefore all robust nodes identified in Table 7 can be reached by means of changes made to management measures. The distance between the reference model parameter value (supposed current “real” state of the system) and the split parameter value identified by the classification tells us how much uncertainty or variability can be tolerated around the reference parameter value to ensure reaching management goals.

**Table 7:** Results of the classification performed with conditional trees for sole.

Leaf 3			Leaf 6						Leaf 10		
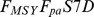 <0.241			**0.241< 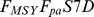 <0.584**						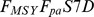 >0.584		
			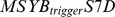 **<0.605**						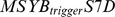 **<0.568**		
			TransitionS7D<**0.75**								
**Subtree 1**			**Subtree 1**			**Subtree 2**			**Subtree 1**		
**Mean Value**	**Split**	**Standard Deviation**	**Mean Value**	**Split**	**Standard Deviation**	**Mean Value**	**Split**	**Standard Deviation**	**Mean Value**	**Split**	**Standard Deviation**
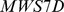 >**0.317**		0.0006	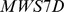 >**0.172**		0.0009	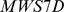 >**0.260**		0.02	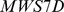 >**0.248**		0.005
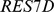 **>0.131**		0.003	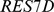 **>0.383**		0.002	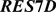 >**0.2 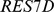 79**		0.02	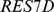 **>0.097**		0.009
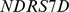 **<0.801**		0.007									

Each block corresponds to a leaf of the main tree and gives conditions on management parameters that are needed to reach it. The lower part of each block corresponds to branches identified from subtrees and gives conditions on environmental parameters that are to be added to those on management to reach a robust terminal node. Cells that are compatible with the reference model parameterisation (i.e. containing value 0.5) are in bold type.

Leaf 6 of the main tree (top center of Table 7) is of particular interest as both management and natural parameter values leading to the robust node are compatible with reference model parameterisation (Table 8). This means that current management measures should allow reaching a spawning biomass of sole above 8000t by 2018, provided reference model parameterisation correctly represents the environment. As our model cannot correctly represent the environment, we look at conditions imposed on environmental parameters by subtree branches. In both cases, only two conditions on environmental parameters are imposed: one on recruitment and the other on mean weight-at-age. The first branch identified tells us that it is possible to reach management goals if (i) mean weight-at-age of sole in area 7D is no more than 32% lower than reference model sole mean weight-at-age and (ii) recruitment until 2018 is no more than 12% lower than recruitment used to perform simulations in our model (which is the geometric mean of recruitment values estimated for the previous years). The second option identified corresponds to a mean weight no more than 24% lower than reference and to a recruitment recruitment no more than 22% lower than reference recruitment value. If a 22% variation around a mean recruitment and a 24% variation in mean weight are deemed sufficient to encompass both natural variability and our uncertainties, then management measures can be left the way they are. If a greater margin is needed on natural parameter values, then management parameters have to be modified.

**Table 8:** Management parameters values identified from leaf 6 and environmental variability they allow dealing with. For sole in subarea 7D.

Management
0.22< 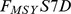 <0.31
0.30< 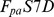 <0.43
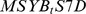 <8840t
TransitionS7D<7.5 yrs
**Environment**
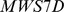 > 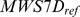 −24%
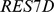 > 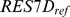 −22%

Moving from leaf 6 to leaf 3 imposes reducing 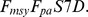 Results for leaf 3 (Table 9) allow greater uncertainties on recruitment than those for leaf 6. In this case, reducing the target fishing mortality of the management measure by 26% or more will allow tolerating recruitments up to 37% lower than the reference recruitment of our model, but lower uncertainties can be tolerated on mean weight-at-age, and natural death rate now also has to be taken into account.

**Table 9:** Management parameters values identified from leaf 3 and environmental variability they allow dealing with. For sole in subarea 7D.

Management
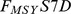 <0.22
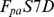 <0.30
**Environment**
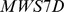 > 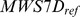 −18%
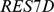 > 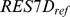 −37%
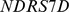 < 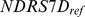 +10%

Results for other leaves of the main tree are rather similar, and correspond to different combinations of the most important management parameters (target F, MSY Btrigger and transition duration). There can be variability in results obtained between leaves, and sometimes stricter conditions on environmental parameters are not observed in leaves where they could be expected (e.g. when the value of FmsyFpaS7D is increased). This illustrates the fact that some results obtained may be too conservative and that some uncertainties remain about the position of the boundary separating acceptable parameter combinations from unacceptable parameter combinations.

#### Plaice 7D

Table 10 shows that, similarly to what was observed for sole, it is the value of fishing mortality targeted by the harvest control rule that mostly determines whether management goals will be reached or not. For plaice, this parameter is associated to the duration of the transition period and to the survival rate of discarded fish.

**Table 10:** Results of the classification performed with conditional trees for plaice.

Leaf 4						Leaf 13						Leaf 12								
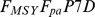 **<0.501**						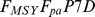 >0.86						0.501< 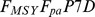 <0.86								
PropSurvP7D<**0.759**						PropSurvP7D>**0.251**						PropSurvP7D>**0.251**								
TransP7D<**0.52**																				
**Subtree 1**			**Subtree 2**			**Subtree 1**			**Subtree 2**			**Subtree 1**			**Subtree 2**			**Subtree 3**		
**Mean Value**	**Split**	**Standard** **Deviation**	**Mean Value**	**Split**	**Standard Deviation**	**Mean Value**	**Split**	**Standard Deviation**	**Mean Value**	**Split**	**Standard Deviation**	**Mean Value**	**Split**	**Standard Deviation**	**Mean Value**	**Split**	**Standard Deviation**	**Mean Value**	**Split**	**Standard Deviation**
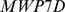 **>0.258**		0.0005	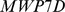 **>0.366**		0.0006	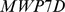 **>0.445**		0.003	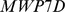 **>0.452**		0.004	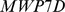 >0.665		0.005	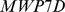 >0.667		0.006	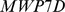 >0.529		0.002
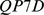 **<0.720**		0.0003	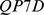 **<0.720**		0.0001	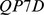 <0.447		0.002	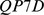 **<0.549**		0.0001	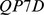 <0.459		0.006	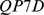 **<0.587**		0.08	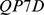 <0.458		0
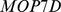 **>0.259**		0.02	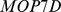 **>0.254**		0.02	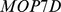 **>0.170**		0.02	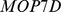 **>0.206**		0.02	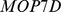 **>0.253**		0.002	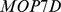 **>0.240**		0.02	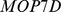 **>0.310**		0.02

Each block corresponds to a leaf of the main tree and gives conditions on management parameters that are needed to reach it. The lower part of each block corresponds to branches identified from subtrees and gives conditions on environmental parameters that are to be added to those on management to reach a robust terminal node. Cells that are compatible with the reference model parameterisation (i.e. containing value 0.5) are in bold type.

Leaf 4 (see Supporting Information S3 for more information about trees built for plaice) if of great interest as it is the only one that contains values of current management parameters, and natural parameters that are compatible with our perception of plaice life cycle. The two options identified in leaf 4 are quite similar, except for the uncertainty that can be tolerated on fish mean weight. This shows that when building trees both split value were identified as having rather similar abilities to split a node into two other nodes, and we cannot say that one value really is better than the other. The goal being to be robust to uncertainties, only the value of 0.366, corresponding to a maximum 13% variability on fish mean weight should probably be considered (Table 11). Uncertainties on parameters such as fish mean weight-at-age or age at maturity usually being lower than uncertainties on recruitment, management goals may still be reachable with the current management (even if the 0.501 threshold on FmsyFpa leaves no room for variation on this parameter, the reference value being 0.5).

**Table 11:** Management parameters values identified from leaf 4 and environmental variability they allow dealing with. For plaice in subarea 7D.

Management
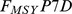 <0.23
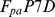 <0.45
TransP7D<5.1 yrs
PropSurvP7D<0.38
**Environment**
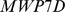 > 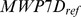 −13%
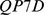 < 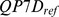 +22%
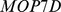 > 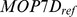 −25%

Values of FmsyFpa above 0.501 (leaf 12 and leaf 13) are associated to survival rates of discarded fish above 0.251, which means that more than 13% of discarded plaice have to survive. Even if this were true, other conditions on natural parameters are not fulfilled except for one branch of leaf 13. This combination of parameters appearing in leaf 13 but not in leaf 12 where management is tighter makes little sense and illustrates tree instability and the need for a bigger training set and a more thorough tree exploration.

Other leaves correspond to other combinations of management and natural parameters. Even if many natural parameters do not agree with our reference model parameterisation, split values for catchability and sometimes fish mean weight are close to model values, and little changes in these parameters could make management goals reachable for a wider range of management scenarios.

## Discussion

### Management Implications

For sole as well as plaice, no combinations of management measures could be identified that always allow reaching management goals accounting for “Nature uncertainty”. However, the sole population in area 7D is in a good enough state to make the 8000 t spawning biomass goal recommended by working groups reachable for a rather wide variety of management parameters values and states of Nature. In particular, management goals on spawning biomass can be reached with current management, provided mean weight-at-age and recruitment of sole do not vary too much. This seems acceptable for mean weight, as it is not a highly variable parameter. On the contrary, variations in recruitment higher than 22% seem likely to happen for sole in the Eastern Channel [55]. In this case, the model suggests strongly reducing the target fishing mortality (division by more than two of 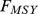 and 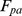) so as to be able to withstand much stronger variations in recruitment.

For plaice in area 7D the spawning biomass threshold chosen by the working group is also Bpa = 8000 t, which corresponds in the evaluation to a fishing mortality threshold Fpa = 0.45. However, this goal is harder to reach for the plaice population in 7D than it was for sole. This can be seen with the much smaller ranges of values of management parameters that allow reaching management goals. However, tolerable ranges of values of natural parameters are rather broad, and these parameters are not known for their high variability (in particular, recruitment is not one of them, and could have been an issue otherwise [56]). Therefore, management goals could be reached, provided plaice stocks are carefully managed. Trees built for plaice are also less stable than those of sole. This instability may have two causes: (i) too few model simulations reach management goals, i.e. the state of the stock is so bad that only a fraction of parameter values tested yield acceptable results or (ii) uncertainties in the life traits of the modelled species are so high that parameters importance and split values cannot be assessed correctly. These two aspects may be linked, bad stock state possibly leading to more variability.

These results are coherent with what is known of the history of sole and plaice stocks in the English Channel. Indeed, mean fishing mortality estimated by working groups for sole remained between 0.3 and 0.6 since 1989 whereas plaice fishing mortality evolved between 0.45 and more than 1.2 (most values being equal to or above 0.6) during the same period [57]. Target and precautionary fishing mortalities for both species being rather similar, it can be said that plaice was more overexploited than sole. Management goals being difficult to reach for plaice our model cannot correctly predict successes, which increases tree instability. As the state of the stock improves, tree stability and therefore our ability to make correct predictions will increase. It is nonetheless worth noting that despite model simplicity and uncertainties our results are coherent with what is known about the studied fish stocks.

Results presented in this article only concern spawning biomass, but similar analyses were performed for the other output variables mentioned previously. The goal here is not to look for the most robust management method, which makes little sense if the analysis is not multivariate. For instance, the best way to maximize spawning biomass is to stop fishing, and there is no need for a complex model to determine this. The interest here is to find management measures that allow reaching management goals on various (and possibly conflicting) output variables and determine how all these constraints on management can be combined. In particular, the key point is to find management measures that allow keeping biomass to acceptable levels while guaranteeing a high enough income to fisheries. A first insight can be obtained by looking for conditions ensuring that catches of flatfish species do not go below a certain level. As it is possible to model fishing costs and fish price in the ISIS-Fish model, it would be possible to look for conditions allowing reaching given economic goals, provided these criteria can be found. Here, we chose to study output variables separately and then look for similarities or discrepancies in management measures manually. But multivariate classification methods exist, and would be an ideal choice here provided hypotheses they are based on and types of results they provide are compatible with our needs. These multivariate techniques could also be used to perform multi species analyses, so that we make sure changing management measures on a species does not negatively impact another species. Here, this problem did not arise since sole is only impacted by “sole” parameters and plaice by “plaice” parameters (Table 8, Table 9, Table 11).

### Caveats

The main limit of techniques we used is that many model simulations are needed. Otherwise reliability of sensitivity analysis results decreases, as large parts of the input space can be left unexplored, and results obtained with classification trees can become highly unstable if they cannot be trained properly. Here, we could perform many simulations because we used a “simple” model (one run takes about one minute on one core), but this method could be harder to apply to a more complex model with longer simulation times. However, the quick increase in available computing power opens very interesting prospects concerning the exploration of complex models input parameters spaces.

Another limit of the method we used is that we built classification subtrees from the main tree obtained when using the whole training dataset. Even if the main tree's type corresponds to the most common tree type identified, it could be interesting to grow subtrees from the leaves of a mean tree that would account for tree variability. This would allow us to take variability on management parameters into account when building subtrees. As standard deviations can also be computed, this would allow us to determine margins of uncertainty around split values. Depending on the needed level of robustness to uncertainties this would permit testing hypotheses and finding areas considered safe at the needed level of confidence. Another possible concern about classification trees is the size of the sub areas they can distinguish in the input parameters space. Would they be able to find “isles of robustness in a sea of non-robustness” ? No extensive tests were run so as to answer this question but we think the method would still be appropriate in this case, even if it may not be the most suitable one. In this case, no constraints should be applied to the tree building process, so simulation time may increase a lot and trees may become much larger than those presented in this paper. This would also make results a lot harder to interpret and translate into applicable management measures.

Concerning the model itself, the weight of the “Other Métiers” group (i.e. métiers not explicitly taken into account in the model) in the sensitivity analysis shows that additional information could have been obtained, had the fishing activity been modelled in more details. This may illustrate the fact that our model is overly simplified and does not take into account enough parameters (at least when modelling the fishing activity). Indeed, we only model explicitly two species and three fleets corresponding to two gear types. Besides, fishing, management and population zones are defined at the scale of the ICES area. Therefore, only large-scale changes can be tested. As few ecosystem compartments are modelled, we only focus on a limited number of processes. Adding information to the model may greatly change its behaviour, especially because of interactions between parameters that cannot be observed in the current simple model. Changing the way in which the model represents the studied processes could also have notable impacts. Indeed, [40] state that not taking model structural uncertainty into account is one of the main flaws in the info-gap approaches that have been implemented so far. The same hypothesis is made in our study, i.e. that parameter variability is more impacting than structural uncertainty. We made this hypothesis mostly because our ISIS-Fish model is based on equations that are commonly used by stock assessment working groups, thus our results should at least be coherent with those given in stock evaluations. Nonetheless, structural uncertainty of our model should (at least partly) be assessed in order to determine whether model structure strongly impacts conclusions drawn, or if they depend (as we supposed in this study) on variations of input parameters.

We use a three-years “forcing” period at the beginning of our model, so as to use available information to better represent the studied ecosystem. This allows us to set management parameters to their real value during that period. We also chose to set recruitment to values estimated by working groups for that period. This could partly explain why recruitment little impacts values of output variables in the sensitivity analysis. Besides, we deal with long-lived species, so there can by a many-years delay between changes in recruitment and the observation of impacts on other parameters caused by these changes. For other biological parameters this forcing period was not used and they therefore are fixed to the calibrated 2008 value, with a 50% level of uncertainty. So on the one hand no uncertainties are associated to recruitment for the first tree years, whereas there is uncertainty in the estimation made by working groups, and on the other hand other biological parameters are given a fixed value with high uncertainties during that period. As all past values can be estimated with some level of certainty, it would seem more logical to force all biological parameters for the first tree years to their estimated values and give them low uncertainty levels corresponding to the accuracy of the estimation. Then for values from 2011 onwards either calibrated or mean values can be used, with much higher levels of uncertainty depending on the level of natural variability and uncertainty corresponding to these parameters.

The question of the range of parameter values to explore is linked with these levels of variability and uncertainty. We chose to perform sensitivity analysis on a 50% window around our model reference parameterisation, while 20% variations are commonly tested when performing sensitivity analyses [58], [59]. As variability can be very high on some parameters and very low on others, this method could be improved. Indeed, it gives too high a weight to parameters the variability of which is overestimated and lessens the weight of parameters with higher variability. A next step in the modelling process is to perform sensitivity analysis on the domain of variation of parameters tested.

Model simplicity and modelling choices we made prevented us from testing spatial management measures. In addition to model changes that we discussed, our goal now is to better represent fish populations and fleets targeting them in our model. In particular, decreasing the scale at which processes are modelled may allow us to test spatial management measures. Combining these measures to those already included in our model, we hope to find out if spatial management measures such as MPAs can allow reaching management objectives with a higher robustness to uncertainties on the state of the ecosystem.

### Management Strategy Evaluation

The approach we used is very close in essence and in goals to that implemented when performing a management strategy evaluation (MSE). Indeed, when performing MSE, the goal is to assess the consequences of a set of management procedures against key performance measures [60]. Similarly to what we wanted to do, the MSE approach does not seek to prescribe an optimal strategy, but rather to provide decision makers with sound information on which to base their decisions. Providing sound information implies identifying strategies that are robust to uncertainty and natural variation [61], as was done in our approach. MSEs can be based on various interacting models, from very simple ones to very complex ones.

The main difference between our approach and common MSE is that we did not just test management scenarios but let parameters controlling the Harvest Control Rule vary within a chosen range of values. Therefore, despite strong environmental drivers in our model (possibly coming from choices made when performing sensitivity analysis), our method is able to find a range of management parameters and values that allow reaching management goals. Therefore, instead of determining whether a scenario is robust or not, we can identify a subspace of management parameters values that are robust to uncertainties. Besides we can also define ranges of variation on natural parameters that can be tolerated and still allow reaching management goals, an information of potentially great importance. Therefore, within the range of management parameters values that can yield robust outcomes, managers can choose the combination of values that seems best depending on their goals and their willingness to take risks.

Such results could be discussed with stakeholders so as to determine the opportuneness of various management measures and to better perceive which situations would lead to failures to reach management or economic goals, and potential solutions to avoid them. This method also allows identifying particular input parameters on which uncertainties should be reduced in priority to allow for better forecast. If the cost of a reduction in uncertainty on some parameters is known (e.g. the price to pay to get additional samples) then it can be balanced against the cost of not robustly reaching management goals and choices can be made depending on managers' priorities. Once these important parameters have been identified an adaptive management procedure [62], [63] targeting them in particular could be set up. An interesting feature of the method is that there is no need to identify a priori sources of uncertainty impacting input parameters. Uncertainty is treated as a whole and identification of sources of uncertainty is only an optional step that can be made by the user.

## Supporting Information

Supporting Information S1 **Dealing with conditional trees instability.**
(TEX)

Supporting Information S2 **Building conditional trees for sole in 7D.**
(TEX)

Supporting Information S3 **Building conditional trees for plaice in 7D.**
(TEX)
